# Application of Porphyrins in Antibacterial Photodynamic Therapy

**DOI:** 10.3390/molecules24132456

**Published:** 2019-07-04

**Authors:** Bamidele M. Amos-Tautua, Sandile P. Songca, Oluwatobi S. Oluwafemi

**Affiliations:** 1 Department of Chemical Sciences (Formerly Applied Chemistry), University of Johannesburg, Doornfontein Campus, P.O. Box 17011, Doornfontein 2028, South Africa; 2 Centre for Nanomaterials Science Research, University of Johannesburg, Johannesburg 2000, South Africa; 3 Department of Chemistry, University of KwaZulu-Natal, Private Bag X 54001, Durban 4000, South Africa

**Keywords:** antibacterial photodynamic therapy, porphyrins, bacteria, light, nanoparticles

## Abstract

Antibiotics are commonly used to control, treat, or prevent bacterial infections, however bacterial resistance to all known classes of traditional antibiotics has greatly increased in the past years especially in hospitals rendering certain therapies ineffective. To limit this emerging public health problem, there is a need to develop non-incursive, non-toxic, and new antimicrobial techniques that act more effectively and quicker than the current antibiotics. One of these effective techniques is antibacterial photodynamic therapy (aPDT). This review focuses on the application of porphyrins in the photo-inactivation of bacteria. Mechanisms of bacterial resistance and some of the current ‘greener’ methods of synthesis of meso-phenyl porphyrins are discussed. In addition, significance and limitations of aPDT are also discussed. Furthermore, we also elaborate on the current clinical applications and the future perspectives and directions of this non-antibiotic therapeutic strategy in combating infectious diseases.

## 1. Introduction

The rapidly growing resistance of bacteria to antibiotics has been regarded as one of the most important clinical challenges facing the entire world today. Antibiotics are traditionally used to control, treat, or prevent bacterial infections. However, in recent years, resistance is becoming more of a problem, especially in hospitals. Improper prescriptions of anti-effective agents in the environment and frequent transmission of microorganisms across the world by people travelling around the globe also constitute a major concern [1]. Several epidemiological reports have shown that bacterial resistance to all known classes of traditional antibiotics has greatly increased in the last 20 years, especially in hospitals rendering certain therapies ineffective [2]. For instance, it was reported that there was a significant increase of methicillin resistant Staphylococcus aureus type t002 isolates from hospitals in the United States in 2013 [3]. In addition, about 10% of patients were appraised by the Centers for Disease Control and Prevention (CDC) who develop a hospital-acquired infection when admitted into hospitals in the United States of America; many of which die because of their infections [4]. The situation is more critical in the developing world where the infection rate is about 75% [5,6]. The statistics for South Africa probably fall somewhere in between the foregoing percentage numbers [7].

However, the recent advances in the search for new antibiotics are not adequate to deal with the growing rate of resistant bacteria strains that have emerged in this century [2]. To limit this emerging public health problem there is a need to develop non-incursive, non-toxic, and new antimicrobial techniques that act more effectively and quicker than the current antibiotics [8,9,10]. One of these methods is antibacterial photodynamic therapy (aPDT) [11,12,13]. Several reports have revealed that aPDT is effective against traditional resistant strains in clinical trials [14,15,16], animal models [17,18], and in vitro [19,20,21]. For example, in vitro tests revealed that aPDT efficiently destroy a clinical isolate of *Pseudomonas aeruginosa,* which is considered as one of the life-threatening nosocomial pathogens [22].

### 1.1. Antibacterial Photodynamic Therapy (aPDT)

Photodynamic therapy is traditionally used against cancer in malignant and non-malignant tumors [23]. The suggestion to use PDT for microbial destruction came into reality in the mid-1990s as bacteria cells replicate at a very high rate, much like those of malignant cells [24]. Antibacterial photodynamic therapy, therefore, is a non-antibiotic process, which produces bacterial cell death in the presence of photosensitizing drugs, light energy of appropriate wavelength, and molecular oxygen [25,26].

#### 1.1.1. Mechanism of Porphyrin Photosensitization: Photophysical and Photochemical Processes

**Figure 1:**
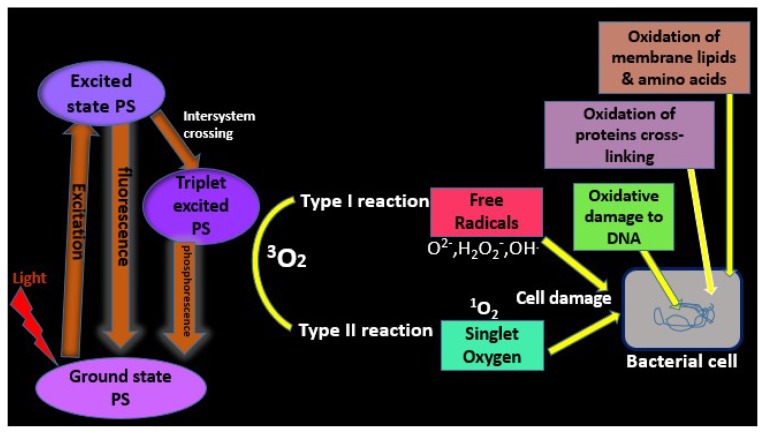
Schematic illustration of photodynamic reaction on bacterial cell [30].

During aPDT, the photosensitizer (PS) is added to a bacterial sample, and the PS-bacteria mixture is irradiated with visible light of a wavelength, which causes excitation of the PS to its singlet excited state. The excited singlet state is unstable and lives for less than 1 μs. It can fall back to the ground state via emission of a secondary photon in the form of fluorescence (Figure 1). The singlet state undergoes intersystem crossing into the excited triplet-state to produce a therapeutic effect [24]. It plays the most significant role in the photochemical reactions involved in the lethal photosensitization (LP) process [27,28]. The PS triplet state can also phosphoresce by obtaining correct spin orientation of its excited electron. It can produce chemical changes in the bacterial cell via two competing pathways, named type I and type II reaction. 

#### 1.1.2. Type I Reaction

This is characterized by dependence on target substrate concentration. The triplet state PS can transfer its energy in the form of an electron or proton to a substrate within the cell (e.g., water in the cell membrane or the cytoplasm), to produce radical ions, which, in turn, react with oxygen to produce cytotoxic reactive oxygen species (ROS) such as superoxide (O^2−^), hydrogen peroxide (H_2_O_2_^−^), and/or hydroxyl radicals (OH^−^). These highly reactive radicals can pass quickly through cell membranes causing oxidative damage and cannot be expelled from the cell. They can also react with organic substrates (e.g., fatty acid, lipids, oxygen) in a series of chain reactions to produce more cytotoxic radicals [29].

#### 1.1.3. Type II Reaction

This is characterized by dependence on oxygen concentration. The triplet state PS can transmit its energy directly to triplet ground state molecular oxygen (^3^O_2_) found in most cells to form excited state singlet oxygen ^1^O_2_ [31]. PDT relies on the production of ^1^O_2_ as the predominant cytotoxic ROS for its lethal effects. It reacts with more than one target within a cell including DNA bases, proteins, and cholesterol found in cell membranes [32].

These ROSs cause bacterial cell death through several mechanisms. These include oxidation of membrane lipids and amino acids in proteins, cross-linking of proteins, and oxidative damage to nucleic acids, which results in the disruption of the normal functioning of the microorganism (Figure 1). This is the principal route of bacterial cell death in vitro [33,34].

The singlet oxygen has been considered the main ROS through which the PS exert their photodynamic action [35,36,37] and it has also been shown to inactivate the antioxidant enzymes such as superoxide dismutase, catalase, and peroxidase [38,39]. 

Many of the PSs such as porphyrins, chlorins, and phthalocyanines utilized for aPDT studies have the macrocyclic tetrapyrrole nucleus, however, porphyrins are the most widely used PDT drugs.

### 1.2. What Are Porphyrins?

**Figure 2:**
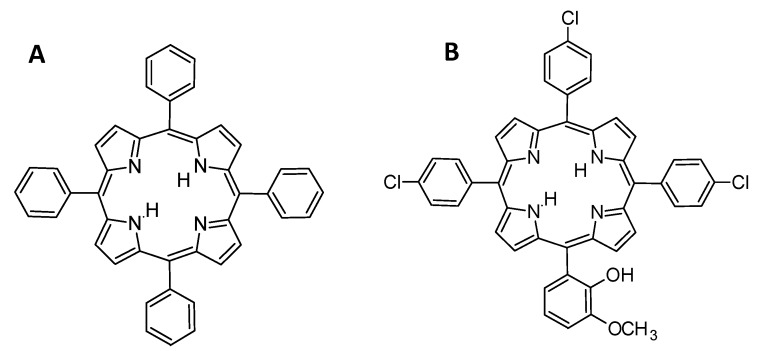
(**A**) Tetraphenylporphyrin and (**B**) 5,10,15-Tris(*p*-chlorophenyl)-20-(2-hydroxy-3-methoxyphenyl)-21*H*,23*H*-porphyrin.

The word “porphyrin” is derived from the Greek word “Porphura” meaning “Purple”. Porphyrins are a large group of fluorescent crystalline intensely colored pigments with natural or synthetic origin [40,41]. They are made up of a substituted aromatic macrocyclic ring consisting of four pyrrole-type residues, connected by four methine groups (Figure 2).

**Figure 3:**
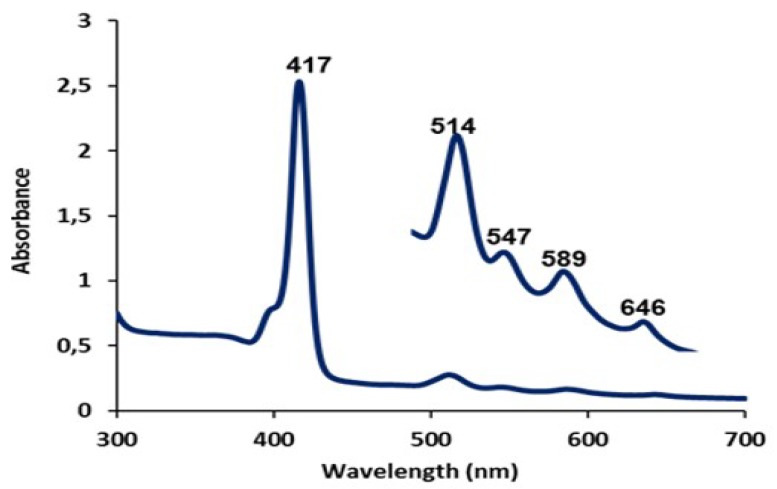
Absorption spectrum of *meso*-5,10,15,20-tetrakis(3,5-dimethoxyphenyl)porphyrin in dichloromethane.

In compliance with Hückels rule of aromaticity (4n + 2 π electrons), the porphyrin nucleus possesses a total of 22 π electrons, with 18 π electrons delocalized over the macrocycle. Due to their aromatic nature, porphyrins generally participate in electrophilic substitution reactions at the meso positions, which are the most electron dense and, as such, are the most reactive [40]. Porphyrins have very intense absorption bands in the visible region due to the extensive electron delocalization within the macrocyclic molecules, which in turn, is responsible for their characteristic bright colors. They show a strong absorption band at ~420 nm, known as the Soret band and the weaker satellite absorption Q bands between 600 nm and 800 nm (Figure 3) [42]. 

#### 1.2.1. Porphyrins as Colors of Life

**Figure 4:**
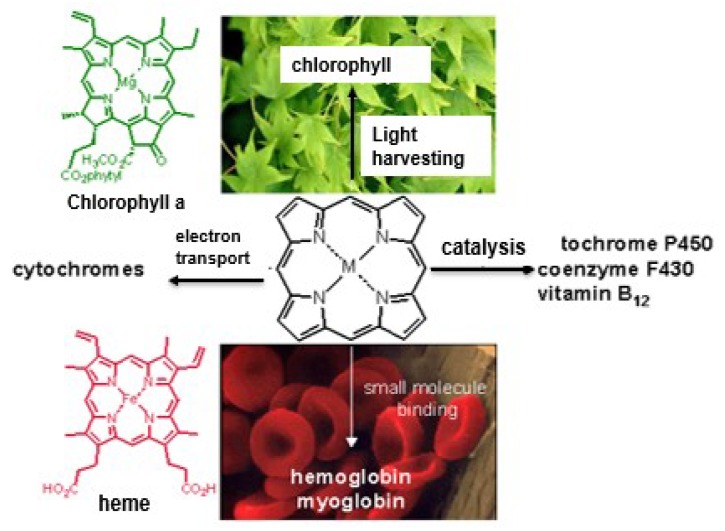
Porphyrins found in nature [43].

Porphyrins are often regarded as colors of life because they are widely distributed in living tissues [40] where they participate in vital biochemical processes (Figure 4), namely the oxygen transport (myoglobin and hem) and the photosynthesis (chlorophylls). They are also involved in electron transport (cytochromes b and c), and O_2_ activation and utilization (cytochrome P450, cytochrome oxidase, and vitamin B12) [32].

#### 1.2.2. Classification of Porphyrins

**Figure 5:**
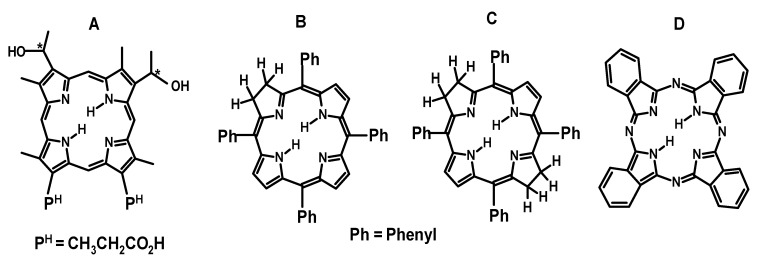
Structures of porphyrin derivatives. (**A**) Hematoporphrin (HpD); (**B**) Chlorin; (**C**) Bacteriochlorin; (**D**) Phthalocyanine [47].

Porphyrins can be classified as first and second generations [44]. The first-generation porphyrins are the primitive porphyrins known as hematoporphyrin derivatives (HpD) present in the first commercially available PDT drug, Photofrin. These porphyrins are limited by impurity, poor in-depth light absorption, and photosensitivity [45]. The latter is an uncomfortable body reaction that occurs because of the activation of the photosensitizer remaining in the body by sunlight after PDT. The second-generation porphyrins, such as chlorin, bacteriochlorin, and phthalocyanine derivatives (Figure 5) emerged to resolve some of the problems associated with the first-generation porphyrins [44,46]. They are characterized with higher purity, better in-depth light absorption, and lesser photosensitivity [46].

#### 1.2.3. General Synthesis of Porphyrins

The synthetic PSs studied in aPDT are based mainly on meso-tetra-arylporphyrins [48]. The popularity of this type of porphyrins results from their simple preparations and potentials toward further chemical transformations. In fact, the synthetic approaches usually involve the condensation of pyrrole with suitable aldehydes in one-flask or two-step one-flask procedures [49]. The wide range of available aldehydes provides porphyrins with different aryl or heteroaryl substituents at the meso-positions. 

##### Rothemund’s Method

**Scheme 1:**
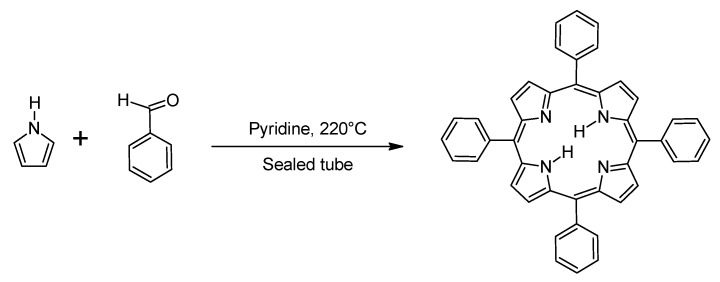
Synthesis of tetraphenylporphyrin via Rothemund method.

Rothemund and Menotti [50] were the first to synthesize meso-tetraphenylporphyrin, thus pioneering the initial synthesis of meso-substituted porphyrins. The compound was obtained by reacting pyrrole and benzaldehyde in pyridine, in a sealed tube at 220 °C for 24 h, with a yield of about 10% (Scheme 1). 

Due to the relatively low yields and harsh reaction conditions, efforts were subsequently geared towards developing more efficient reaction conditions that would enable the preparation of a wider variety of meso-substituted porphyrins with higher yields.

##### Adler–Longo’s Method

**Scheme 2:**
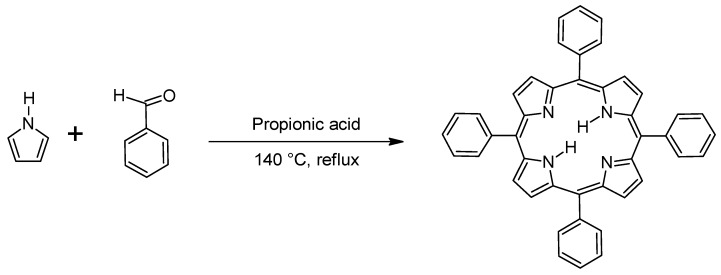
Synthesis of Tetraphenylporphyrin via Adler–Longo’s Method.

Adler and Longo [51] later modified the Rothemund and Menotti method under milder reaction conditions. The reaction modification involved refluxing pyrrole and benzaldehyde in propionic acid for 30 mins at 141 °C (Scheme 2). This affords many more substituents on the benzaldehyde to be converted to their corresponding porphyrins in yields of up to 20%. The disadvantage of this protocol is that purification of the product is tedious due to the production of a high degree of tar during the reaction. Also, attempts at making porphyrins from benzaldehydes bearing sensitive functional groups such as hydroxyl, thiol, and amino groups failed under these reaction conditions. 

Regardless of these issues, the relative simplicity and robustness of Adler–Longo method is still regarded as the most effective in the large-scale preparation of porphyrins. 

##### Lindsey’s Method

**Scheme 3:**
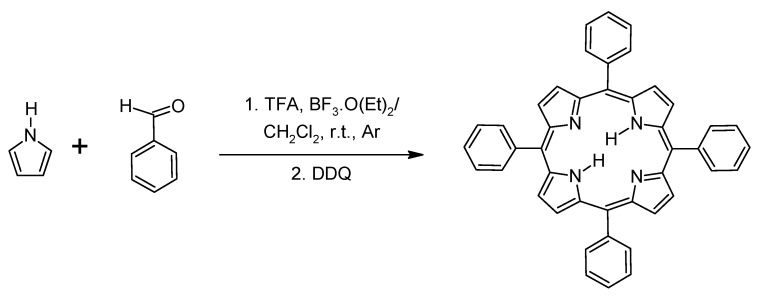
Synthesis of meso-tetraphenylporphyrin via Lindsey’s Method.

Lindsey and co-workers [49] developed a protocol that can be utilized to make porphyrins that require the use of acid-unstable aldehydes not generally used under Adler–Longo method. In the Lindsey reaction, equimolar concentrations of pyrrole and benzaldehyde are reacted with trifluoroacetic acid (TFA) or boron trifluoride etherate (BF_3_.OEt_2_) as catalysts, at room temperature, under an inert gas atmosphere for 1 h in dichloromethane (DCM) as solvent, using a water scavenger (triethyl orthoacetate). This is followed by the addition of 2,3-dichloro-5,6-dicyanobenzoquinone (DDQ) to convert the prophyrinogen intermediate to porphyrin (Scheme 3). The advantages of this method are that it allows more facile purification and affords higher yields.

##### Other Synthetic Methods

There is a growing need for the development of new synthetic routes involving environmentally sustainable processes, hence, some strategies were recently adopted to prepare meso-substituted porphyrins using alternative energy sources, reaction media, and catalysts, including microwave irradiation, water as solvent, or solid acid catalysts. These synthetic methods are discussed in the following sections.

##### Microwave-Assisted Synthesis

**Scheme 4:**
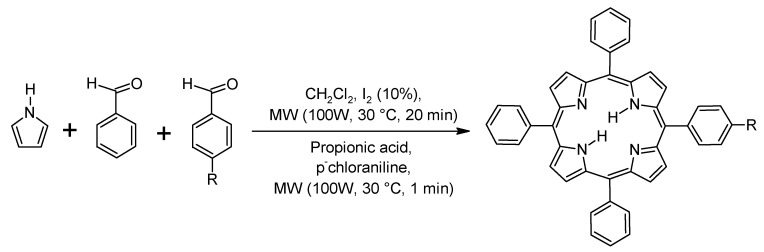
Synthesis of meso-substituted porphyrins under microwave irradiation.

Zerrouki et al. [52] employed microwave-activated two-step synthetic approach to prepare some unsymmetrical A_3_B type meso-tetraarylporphyrins. Benzaldehyde, pyrrole, dichloromethane, and a 10% molar equivalent of molecular iodine were activated under microwave (MW) irradiation, then p-chloraniline was added and a second session of microwave irradiation was effected (Scheme 4).

To avoid inconvenient conditions, such as the use of propionic acid, catalytic amount of iodine was used as an acid promoter. The advantages of this protocol over other methods are that it uses reagents and solvents without the need for prior distillation, it is very easy to perform, and gives the final meso-tetraphenylporphyrin product within a short reaction time with reasonable yields (47%). 

**Scheme 5:**
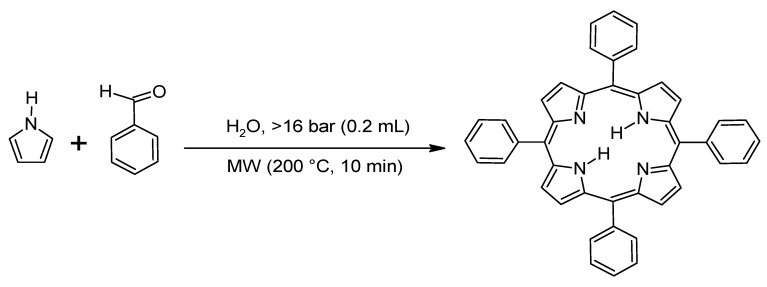
Meso-substituted porphyrin obtained using water as solvent/oxidant microwave irradiation.

Water has been used as potential solvent for hydrophobic organic acid or as base catalyst and, in some cases, as oxidant because of its stability at temperatures above its boiling point and pressures above 16 bar [53]. These unique properties of overheated water under microwave irradiation have been employed for the preparation of meso-arylporphyrins. Henriques and co-workers [54] reported good to moderate yields of porphyrins under these reaction conditions (Scheme 5).

**Scheme 6:**
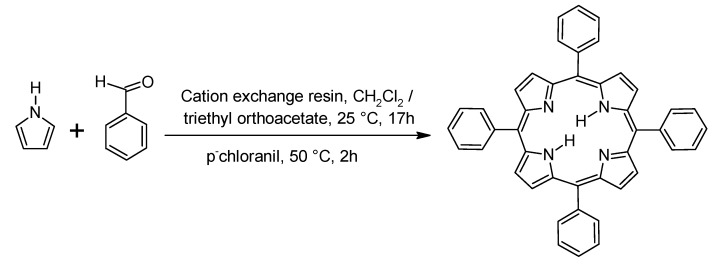
Synthesis of meso-substituted porphyrins using solid acid catalysts.

In another development, Joshi [55] described a ‘greener’ approach method for the synthesis of meso-arylporphyrins, in which the obnoxious strong organic acids (TFA, TsOH, and BF_3_∙Et_2_O) are replaced by the acidic cation exchange resin Indion-130 (Scheme 6). The resin was used as a catalyst in the condensation of pyrrole with several substituted aromatic aldehydes, using triethyl-orthoacetate and dichloromethane as solvent.

##### Solventless Reactions

**Scheme 7:**
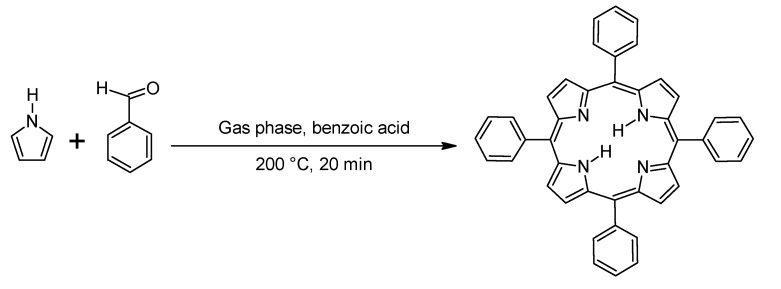
Synthesis of meso-substituted porphyrins using solventless reaction conditions.

Drain and coworkers [56] developed a proficient one-step route for the synthesis of meso-tetraarylporphyrins using only oxygen as oxidant. Benzoic acid was added as adjuvant to the reaction mixture of pyrrole and benzaldehde (in gas phase) to improve the yield of the porphyrin (Scheme 7). 

##### Ionic Liquids as Alternative Solvents

**Scheme 8:**
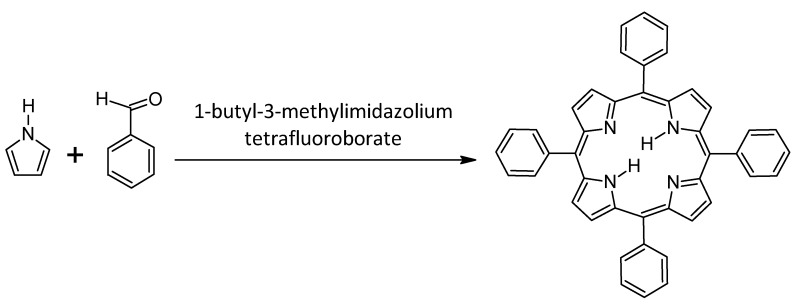
Synthesis of meso-substituted porphyrins involving the use of ionic liquids.

Chandramouli [57] reported a fast and efficient pathway for the synthesis of meso-tetra-arylporphyrins, using an acidic ionic liquid, 1-butyl-3-methylimidazolium tetrafluoroborate ([bmim]BF_4_) as solvent to catalyze the condensation of pyrrole with aryl aldehydes under Adler’s reaction conditions (Scheme 8). The products were obtained in higher yields than those of the traditional Adler’s method.

### 1.3. Unique Properties of Porphyrins as Drugs for aPDT

Many groups have shown porphyrins to be efficient PSs for use in aPDT. Porphyrins have demonstrated significant broad spectrum of action against Gram-(+) and Gram-(−) bacteria at very low concentrations (0.1–5 μM). Their antibacterial activity induced photodynamic therapy shows the following unique properties.

- Porphyrins have relatively low toxicity in vitro and in vivo and can be functionalized to be water soluble or water insoluble [58].
- They can be cleared in a reasonable time from the body and rapidly from the skin to avoid photosensitive reaction [32].
- Porphyrins can also possess competent amphiphilicity and ability for numerous chemical modifications [59].
- They have high quantum yield (above 0.70) for ^1^O_2_ generation and high one-photon absorption coefficient (≈500,000 M^−1^cm^−1^) [60].
- Porphyrins possess a high binding affinity to cellular components, membranes, proteins, and DNA [61,62].
- Furthermore, they have a “therapeutic window” whereby they are active in killing bacterial cells, including multi-drug resistant bacteria (e.g., MRSA), whilst not damaging cultured human cells [11,63].
- Porphyrins possess large number of different mechanisms in affecting microbial and viral pathogens. The possibility of bacteria acquiring resistance will be sufficiently reduced [1,64].

## 2. Bacteria

Although some bacteria are vital for our everyday health and wellbeing others can also cause infection, hence, methods of eradicating virulent bacteria are needed. Bacteria are generally classified into two groups; namely Gram-(+) such as *Staphylococcus aureus, Bacillus subtilis, and Enterococcus faecalis* and Gram-(−) such as *Escherichia coli, Pseudomonas aeruginosa,* and *Klebsiella pneumonia* [65].

### 2.1. Differences in Membrane Structure Between Gram-(+) and Gram-(−) Bacteria

**Figure 6:**
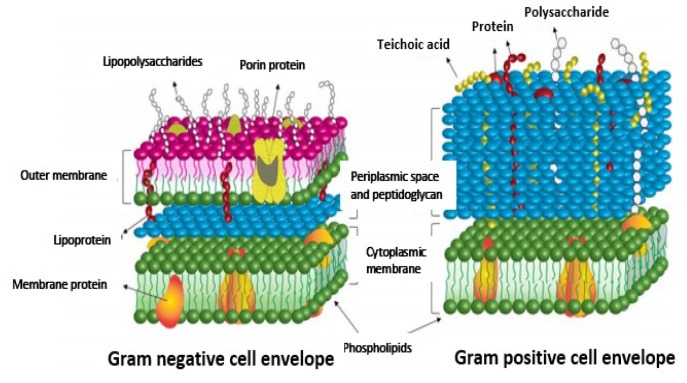
Schematic representation of cell wall and cytoplasmic membrane structure in Gram-(−) (left) and Gram-(+) (right) bacteria [12].

Differences in the cell wall ultra-structure of Gram-(+) and Gram-(−) bacteria (Figure 6) play a remarkable role in the sensitivity of bacteria to LP. Generally, all classes of PS molecules bind efficiently to Gram-(+) bacteria and inactivate them but Gram-(−) bacteria are known to be more resistant to treatment with PS [66,67]. 

The high susceptibility of Gram-(+) species to PS is attributed to the presence of a relatively porous layer of peptidoglycan and lipoteichoic acid in their cell wall, which allows PS molecules to diffuse to the target sites within the cell. In contrast, the cell wall of Gram-(−) bacteria contains negatively charged lipopolysaccharide (LPS), which hinders the permeability of neutral or anionic porphyrins in the external environment into bacterial cell; however, cationic porphyrins interact effectively with these negatively charged surfaces of Gram-(−) bacterial cell wall and photo-inactivate them [2,11,68]. 

There are two approaches to overcome this problem and achieve broad spectrum activity especially with neutral and anionic PSs. The first approach is to use membrane disorganizing agents to enhance their permeability such as polymyxin B nonapeptide (PMBN) or ethylenediaminetetraacetic acid (EDTA) [69]. These agents destabilize the cell wall structure by removing the Mg^2+^ and the Ca^2+^ ions, which neutralize the superficial negative charges. Malik and co-workers [70] found that pre-treating bacterial cells with EDTA results in the loss of a substantial amount of their LPS. Cells pre-treated with PMBN did not cause the release of LPS into the medium as with EDTA. The polycation displaces the divalent cations by binding strongly to the highly negatively charged surface. This causes an expansion on the surface of the outer membrane and allows the diffusion of hydrophobic molecules. Thus, the use of PMBN and EDTA widens the spectrum of photodynamic inactivation of bacteria.

The second approach is to attach a cationic polypeptide to the neutral or anionic PS molecule, so that it can bind to the negative charges of LPS [71,72].

Another solution is increasing the selectivity of the PS to the target micro-organism [73]. This can be accomplished through the conjugation of the PS molecule to monoclonal antibodies or bacteriophages, which allow selective binding to specific structures of the target microorganism. This approach can limit the destruction to host tissues surrounding the infected area. These techniques have been verified successfully in vitro against MRSA [48,74,75] and in vivo against a *P. aeruginosa* skin-infection model in mice [20,76,77].

#### 2.1.1. Mode of Porphyrin Action on Bacterial Cell

Larson and Marley [78] have described three modes of action by which light-activated antimicrobial agents can interact with the cell:

1. The first is that the PS settles outside the cell, generating reactive oxygen species in solution, which can diffuse into the cells of the target organism and react to induce cellular damage.
2. The second mechanism is that the PS binds to or becomes localized at the cell membrane (by hydrophobic or coulombic interactions)—upon light absorption, the PS transfers energy (e.g., an electron, hydrogen atom etc.) to target biomolecules within the cell, resulting in ROS production that cause cell damage. Anionic porhphyrins follow this mechanism of photosensitization.
3. The third possibility is that the PS penetrates the interior of the cell and becomes associated with an intracellular target, possibly a protein (inducing enzymatic damage) or the nucleus (inducing genetic damage). Cationic porphyrins that bind strongly to the polyanionic macromolecules such as DNA are good examples of this type of phototoxic agent [79].

#### 2.1.2. Mechanisms of Porphyrin Photodynamic Inactivation of Bacteria

The mechanisms by which porphyrins cause bacterial cell death are complex and non-specific. Many investigators [37,68,80,81,82] have suggested that the ROS generated from type I and II reactions can cause microbial cell damage via three main mechanisms.

##### Functional Damage

These comprise the inactivation of essential enzymes, oxidation of protein−protein cross−links, and inhibition of metabolic processes such as DNA synthesis, and glucose transport [11,34].

##### Morphological Changes

These include alteration of mesosome structure [83]. Mesosomes are invaginations of the plasma membrane responsible for the synthesis of cross-wall in dividing bacteria. They are usually tubular, vesicular, or lamellae in shape [84]. When porphyrins bind to the plasma membrane of bacteria, the structure of mesosomes are altered and they take a laminated, myelin-like form, which subsequently disrupt the division of bacterial cells.

##### Cell Membrane Damage

This may result in the breaking down of cellular contents and subsequent disruption of the membrane transport system and enzymes [85,86].

Although some damages to the nucleic acid may occur, it has been discovered that these damages can be repaired by DNA repairing systems [87,88].

## 3. Choice of Light Source

The choice of light source to photo-inactivate pathogens depends primarily on the depth of the infected tissues into which the light should penetrate. The selected wavelength must match the absorption spectrum of the PS of choice. The most effective irradiation is that in the red and NIR range of the spectrum [89]. 

**Figure 7:**
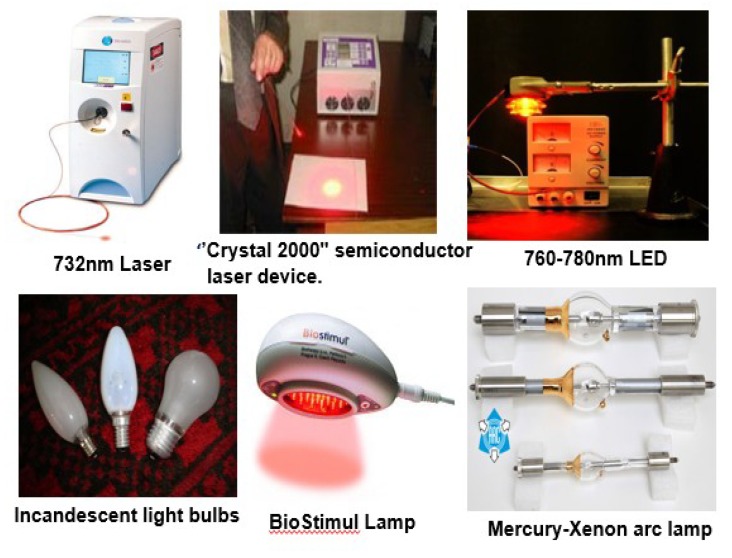
Examples of light sources [92].

A few collections of light sources (Figure 7) have been used over the years. The most popular laser being the Argon dye laser, with tuneable wavelength that is suitable to that of the optimum absorption wavelength of the PS. However, they are costly, need an external water-cooling system and separate power supply, as well as a lot of maintenance [90].

The He–Ne laser and the semiconductor diode lasers are two laser systems that have been used widely for photodynamic inactivation of bacteria in recent years. They are cost effective because of their small size, handiness, dependability, and reasonably cheapness. However, the wavelength generated is not tuneable, hence, has to be pre-matched for a chosen PS [91]. The He–Ne laser radiates at 632.8 nm while other semiconductor diode lasers emit at longer wavelength of 630–950 nm.

Non-laser light sources are used primarily in dermatology. These sources are regular lamps that produce incoherent light, generate a lot of heat, whose output wavelength is achieved by using filters [93]. There are many varieties of lamps that emit incoherent light with continuous spectrum such as incandescent lamps, xenon arc lamps, and those with the spectrum in bands (gas discharge lamps or metallic vapor lamps), which have been used to lethally photo-inactivate some of the pathogens associated with wound infections [91].

For example, a halogen lamp with a wavelength range between 350–800 nm at an intensity rate of 90 mW/cm^2^ in combination with porphyrin has been used successfully to inactivate *E. coli* [94]. 

However, for in vitro aPDT studies, broadband light sources such as broad arrays of light-emitting diodes (LED) are considered suitable light sources [95]. LED are preferable than other light sources because of their large output, lower thermal tissue destruction, facile fabrication, large area illumination, and cost efficiency [96].

## 4. Antibacterial Photodynamic Effects of Porphyrins

Many groups have shown porphyrins to be efficient PSs for use in aPDT. Studies by Orenstein et al. [97] showed that it was possible to kill *Staphylococcus aureus,* a Gram-(+) bacterium, using deuteroporphyrin but Gram-(−) bacteria such as *Escherichia coli* and *Pseudomonas aeruginosa* could not be inhibited using deuteroporphyrin alone. Malik et al. [70] overcame this problem by pre-treating the cells with either EDTA or PMBN. 

Other studies, conducted using meso substituted porphyrins, have also demonstrated that the molecules need to be cationic in order to photoinactivate both Gram-(+) and Gram-(−) bacteria. For example, Ragas and co-workers [82] investigated the potential of aryl cationic porphycenes as photosensitizing drugs in aPDT in vitro and in vivo infection models and noticed that the structural porphyrin isomer successfully inactivated different Gram-(+) and Gram-(−) bacterial and fungal cells. Zoltan et al. [98] also observed an efficient inactivation of *Escherichia coli* when meso-tetra(pyren-1-yl)porphyrin complexes of Ni(II), Cu(II), and Zn were tested against the bacteria.

Tavares et al. [99] examined the mechanisms of photodynamic inactivation (PDI) of bacteria using cationic porphyrins. They proposed that singlet oxygen is more responsible for the PDI process of the bioluminescent *E. coli* than free radicals generated by the cationic porphyrins. 

Maisch et al. [100] studied the inactivation mechanism of several cationic porphyrins against different *S. aureus* and *E. coli* strains and concluded that the killing was mediated predominantly by ROS, including singlet oxygen. Furthermore, a novel porphyrin-based photosensitizer, XF73, has been reported by Taub et al. [101] to show high efficacy in killing MRSA without damage to healthy human cells. They concluded that this PS can inhibit MRSA infection in hospitals as well as for burns and other open wounds.

Nitzan and Ashkenazi [102] revealed the effects of illumination by different light sources at different wavelengths in *Dinococcus radiodurans,* using two different porphyrin derivatives; the hydrophilic cationic 5,10,15,20-tetrakis(*N*-methylpyridinium-4-yl)porphyrin (TMPyP) and the neutral derivative deuteroporphyrin (Dp). The most potent photo-destruction were obtained for both photosensitizers when *D. radiodurans* cultures were treated with blue light (400–450 nm). Blue, green, and red light were also used to induce photoinactivation of multiple antibiotic-resistant bacteria *Acinetobacter baumannii* and *Escherichia coli* by the cationic photosensitizer and similar results were obtained. Complete extermination of both bacteria was obtained with blue light [103]. 

Several studies have been conducted using (5,10,15,20-tetrakis(*N*-methylpyridinium-4-yl)porphyrin. For instance, Maisch et al. [104] found that cells of *Vibrio fischeri* and of *Escherichia coli*, and T4-like phages, that were photoinactivated in vitro to the detection limit in the presence of 5,10,15,20-tetrakis(*N*-methylpyridinium-4-yl)-20-(pentafluorophenyl)porphyrin triiodide do not recover their viability in laboratory conditions after one week.

**Figure 8:**
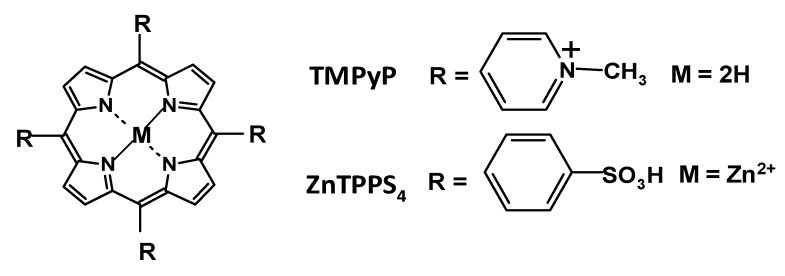
Structures of TMPyP and ZnTPPS_4_.

Hanakova et al. [105] examined the antibacterial PDT effect of TMPyP (5,10,15,20-tetrakis(*N*-methylpyridinium-4-yl)porphyrin and ZnTPPS_4_ (Zinc-5,10,15,20-tetrakis(4-sulphonatophenyl) porphyrin (Figure 8) bound to hp-β-cyclodextrin. They found that both porphyrins were able to reduce the growth of *S. aureus* and *E. coli* strains 

In a recent development, Ruiz-Gonzalez and co-workers [106] used the novel tricationic porphycene,2,7,12-tris(trimethyl-*p*-tolyl)–17-(*p*-(methoxymethyl)phenyl)porphycene (NMe_3_MeO-TBPo,) and its predecessor, 2,7,12-tris(α-pyridinio-*p*-tolyl)–17-(*p*-(methoxymethyl)phenyl) porphycene (Py_3_MeO-TBPo,) to photoinactivate Gram-(+) *S. aureus* and Gram-(−) species such as *P. aeruginosa* and *E. coli.*

**Figure 9:**
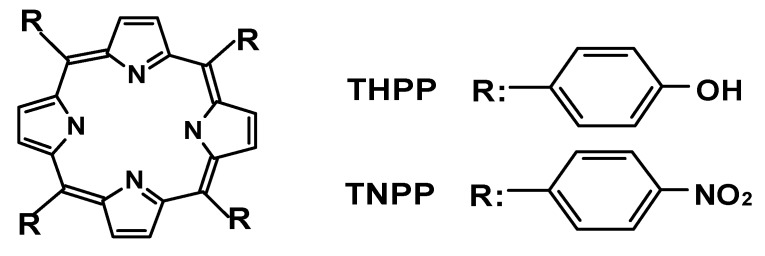
Structures of neutral porphyrins.

Neutral porphyrins like 5,10,15,20-tetrakis(4-hydroxyphenyl)-porphyrin (THPP) have been considered as less phototoxic towards Gram-(−) bacteria. However, Wikene et al. [107] had shown that only nanomolar amounts of THPP (Figure 9) in natural deep eutectic solvents (NADES) were needed for complete photoinactivation of *Enterococcus faecalis* and *Escherichia coli*.

Rahimi et al. [108] also investigated the effect of 5,10,15,20–tetrakis(4-nitrophenyl)porphyrin (TNPP) and its zinc derivative on photoinactivation of *P.aeruginosa* and *B. subtilis.* The results show that photoactivated TNPP (Figure 9) and ZnTNPP have effective inhibitory activity against *B*. *subtilis* and *P. aeruginosa*, as compared with ampicillin. The interesting thing about TNPP and ZnTNPP antibacterial activity is that it seems to be more active against the Gram-(−) *P*. *aeruginosa*, than the Gram-(+) *B. subtilis*. 

## 5. Significance of aPDT

Antibacterial effectiveness of PDT is considered a promising alternative to other kinds of antibiotic treatment for several reasons, which include its multi-target process, broad spectrum of action, broader therapeutic window than other antimicrobial therapies, even against pathogenic biofilms. Because of the high reactivity of ROS, secreted virulence factors can be destroyed as these are commonly proteins, enzymes, or amino acid residues [63]. aPDT is independent towards the resistance pattern of bacteria to antibiotics; it produces extensive bacteria reduction with restricted damage to healthy host tissue, and specifically delivers PS to the infected area [81,83]. It demonstrates fast inactivation than usual antimicrobials [11,99,109,110]. It shows absence of photo-resistant strains and viability recovery after multiple treatments [80,111,112,113]. It requires low infrastructure and equipment [114]. It is a simple-to-use and cost-effective therapeutic option for developing countries [115].

## 6. Clinical Applications of aPDT

Many clinical trials of porphyrins and aPDT have been carried out for the treatment of dermatological conditions [116,117,118]. Moreover, their intensive use in periodontitis [15,16,119,120] has opened a wider application in the treatment of other infectious diseases of microbial origin. It is currently envisaged that porphyrins would have application for treatment of topical human infections and to replace ‘skin applied’ antibiotics [121,122].

### 6.1. Treatment of Wound Infections

**Figure 10:**
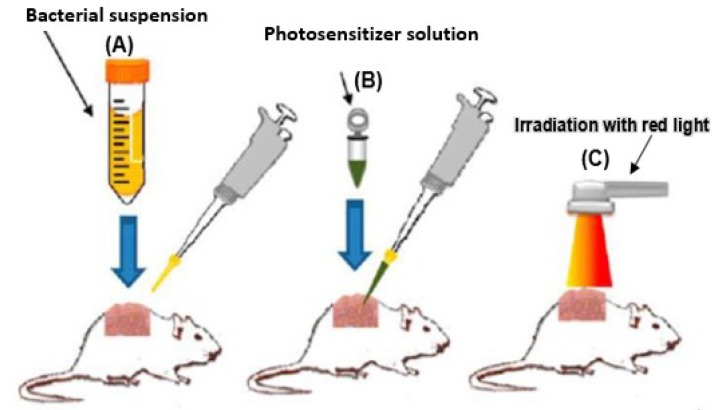
Schematic diagram showing the stages involved in carrying out antimicrobial PDT on a burn infection in mice. (**A**) Inoculation of the mouse with bacterial suspension; (**B**) injection with photosensitizer solution; (**C**) irradiation of the infected site with red light [11,77].

A wound is a breach of the skin that can lead to infection and sepsis. When the bacterial load exceeds 10^5^ organisms per gram of tissue, or when the immune system becomes suppressed, infection develops [123]. Hamblin and co-workers [11], using an animal model, demonstrated that bacteria infecting a wound could be destroyed photo chemically without affecting the healthy host cells. (Figure 10). *E. coli* present in the wound were quickly annihilated when chlorin(e6) photosensitizer conjugated with poly-l-lysine, was topically applied. Likewise, porphyrins showed to be very effective in destroying of *S. aureus* and selected viral pathogens in burn wound infections [17,124,125]. Barra et al. [126] explored the possibility of fighting biofilms produced by the Gram-(+) bacteria *Staphylococcus* species that commonly crowd superficial wounds and sores by combining PDT using 5-aminolevulinic acid with an antibiotic, gentamicin. It was observed that the combined treatment effectively caused detachment of the biofilm and bacterial death. Research findings have shown that PDT has the capacity to destroy secreted virulence factors such as protease, which was speculated to be responsible for healing in *P. aeruginosa*-infected wounds [76,77].

Recently, Bartolomeu [127] and her co-workers assessed the impact of photodynamic therapy (PDT) on the virulence factors of six strains of *S. aureus* using the photosensitizer (5,10,15,20-tetrakis(*N*-methylpyridinium-4-yl)porphyrin tetraiodide. They found out that the expression of some external virulence factors was affected by antimicrobial photodynamic inactivation (PDI) and enterotoxin producing strains were more susceptible to PDI than non-toxigenic strains. The result was that the surviving bacteria did not develop resistance to PDI treatment.

### 6.2. Treatment of Acne

Acne is the most prevalent skin disease. It is a condition that occurs when hair follicles become clogged with oil (sebum) and dead skin cells. The commensal bacterium *Propionibacterium acnes* accumulate in these sebaceous follicles where they secrete enzymes that break down sebum and cause inflammatory acne lesions [128,129].

**Figure 11:**
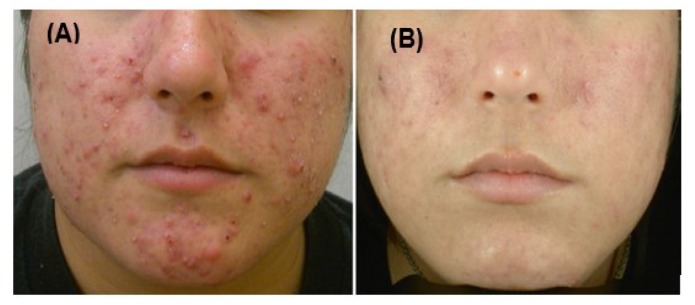
Acne treatment with blue light and endogenous, 5-aminolevulinic acid (ALA)-induced porphyrin. Before treatment (**A**); after six months of therapy (**B**) [89].

Meffert et al. [130] first reported the successful treatment of acne with visible light. Improvement on this therapy was achieved when the endogenous porphyrins of *P. acnes* was photo-inactivated with blue light (Figure 11) [131,132]. It was conceived that porphyrins through the generation of the cytotoxic singlet oxygen might boost the perifollicular inflammatory reaction and activate the expression of keratinocyte-derived IL-8 [133,134].

### 6.3. Periondontal Diseases

Dental infections are the greatest expanding field of clinical antibacterial PDT. Periodontal disease is caused by a set of pathogenic bacterial species that form plaques and accounts for periodontal inflammation and destruction. In vitro studies have revealed that many pathogens such as *P. gingivalis, Fusobacterium nucleatum, and Staphylococcus sp,* which are prevalent in the subgingival periodontal plaques, have been effectively exterminated by photodynamic treatment, both in aqueous suspension and as a biofilm [135]. In addition, Garcia et al. [136] observed that photodynamic therapy causes the reduction of periodontal tissue damage when compared to other treatment methods, such as scaling and root planning and antibiotic therapy.

**Figure 12:**
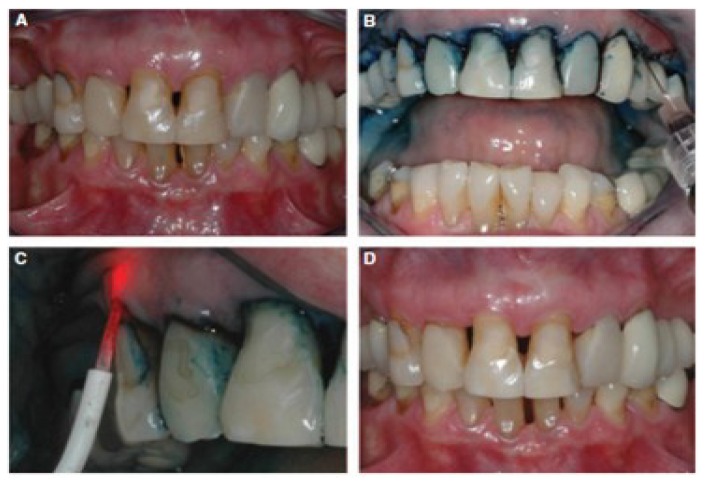
(**A**) Clinical situation of a patient before antibacterial photodynamic therapy (aPDT). (**B**) Injection of the photosensitizer. (**C**) Irradiation with the diode laser. (**D**) The clinical situation six months after therapy [137].

The process of aPDT treatment of periodontal disease may involve injecting a porphyrin into the periodontal pocket, followed by irradiation with fiber optics inserted into the infected area (Figure 12). This method ensures that only the disease lesions are treated while other beneficial microflora remain unaffected. Likewise, Streptococci such as *Fusobacterium nucleatum* and *Actinomyces viscosus,* which causes dental caries, could also be attacked [11].

In a recent development, Cieplik et al. [138] evaluated the ability of (5,10,15,20-tetrakis(*N*-methylpyridinium-4-yl)porphyrin tetra-(*p*-toluenesulfonate) and Methylene Blue to inactivate root canal bacteria, *Enterococcus faecalis.* They observed that photoinactivation of bacteria (PIB) with both PS led to reduction by ≤5 log10 of *E. faecalis* CFU for each setup. Conclusively, they suggested that light activation of given intra-canal PS from outside a tooth may be possible at wavelengths ≤430 nm, facilitating clinical application of PIB in endodontics.

### 6.4. Treatment of Environmental Waters

**Figure 13:**
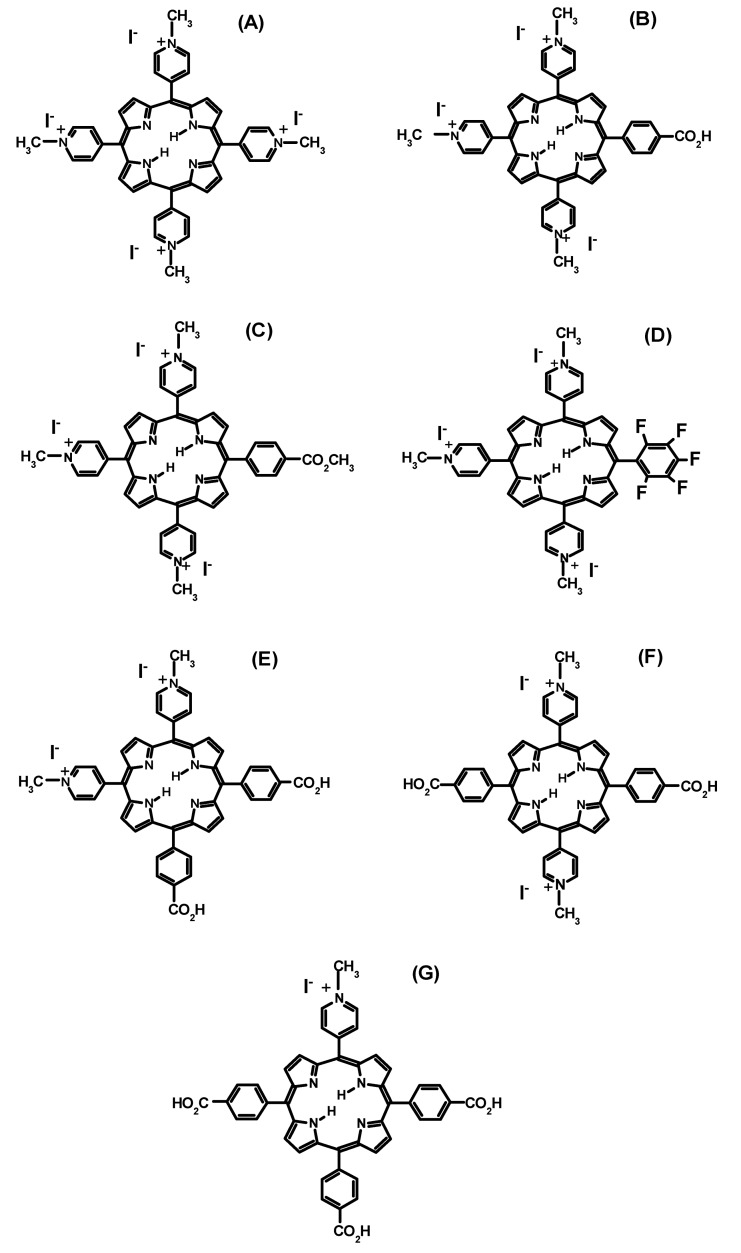
Structures of some cationic porphyrin derivatives. (**A**) Tetra-Py^+^Me; (**B**) Tri-Py^+^-Me-CO_2_H; (**C**) Tri-Py^+^-Me-CO_2_CH_3_; (**D**) Tri-Py^+^-Me-PF_4_; (**E**) Di-Py^+^-Me-Di-CO_2_H adj; (**F**) Di-Py^+^-Me-Di-CO_2_H opp; (**G**) Mono-Py^+^-Me-Tri-CO_2_H; [142].

Several studies have shown that aPDT is effective in the selective inactivation of pathogens in waste waters, hence it is being considered as an alternative for the treatment of environmental waters such as surface, ground, drinking, and waste waters [139,140,141]. This is demonstrated by the fact that Alves and colleagues [142] compared the efficiency of seven cationic porphyrins differing in meso-substituent groups, charge number, and charge distribution, (Figure 13) on the photodynamic inactivation of a Gram-(+) bacterium (*Enterococcus faecalis*) and a Gram-(−) bacterium (*Escherichia coli*). Some of the selected porphyrins have been shown to be efficient PS against other microorganisms such as sewage bacteriophage [143], bacterial endospores [144], sewage fecal coliforms [141], and recombinant bioluminescent *E. coli* [145]. Their results revealed that the various changes in the structures of the porphyrins had different effects on the photoinactivation of both bacteria. They concluded that the most active Tri-Py^+^-Me-PF_4_ porphyrin could serve as efficient photosensitizer that can efficiently destroy a large spectrum of environmental bacteria. This photodynamic approach shows that the application of porphyrins irradiated with natural light sources is cost effective and feasible. 

Almeida et al. [146] evaluated synergistic effect of PDI and antibiotics (ampicillin and chloramphenicol) on four multidrug-resistant (MDR) bacteria (*E. coli, P. aeruginosa, A. baumannii,* and *S. aureus*) in hospital wastewaters. A reduction of 6–8 log of the MDR bacteria was observed after 270 mins of irradiation with white light at 40 W m^−2^ with 5.0 μM of the cationic porphyrin (5,10,15,20-tetrakis(*N*-methylpyridinium-4-yl)porphyrin (Tetra-Py^+^-Me). The presence of the antibiotics significantly enhanced the effectiveness of the PDI of the bacteria.

In another development, Alves [147,148] and coworkers used 5-pentafluorophenyl-10,15,20-tri(4-pyridyl)porphyrin and the corresponding cationic 5,10,15-tris(1-methylpyridinium-4-yl)-20-(pentafluorophenyl)porphyrin tri-iodide immobilized on cationized silica-coated magnetic nanoparticles of Fe_3_O_4_ and CoFe_2_O_4_ for water disinfection. Their results indicated that the cationic nanomagnet-porphyrin hybrids are highly efficient in bacterial destruction in water and wastewater treatment and support many photoinactivation cycles.

## 7. Other Applications of Porphyrins

Several porphyrins are now approved for a variety of diseases, including cancers [149,150,151] and age-related macular degeneration [152,153].

Porphyrin-based compounds have received significant importance in molecular electronics and supramolecular building blocks. Recent applications of porphyrin dyes for dye-sensitized solar cells have shown solar conversion efficiencies in silicon based photovoltaic devices [154,155].

In addition, porphyrins are frequently used to build structures in supramolecular chemistry. For instance, a porphyrin–fullerene complex was involved in host–guest chemistry [156]. Vinodh et al. [157] reported recent developments in the synthesis of porphyrin assemblies associated with cyclodextrins, calixarenes, and resorcinarenes and their potential applications in the fields of molecular encapsulation/recognition, and chemical catalysis.

Also, porphyrins such as nickel and vanadyl porphyrins have been used to establish the biological origins of petroleum in organic geochemistry [158,159].

## 8. Side-Effects and Drawbacks of aPDT

PDT has many side effects and drawbacks. These include optical absorption limitation. Light generated from standard laser and low-powered LED technology cannot penetrate more than 1 cm of tissue depth when it is used to activate photosensitizers. Thus, these light sources are less effective in treatment of systemic infections such as bacteremia and sepsis [160]. Another side effect of aPDT is post-therapeutic photosensitivity [45,161]. It cannot be used in patients sensitive to porphyrins and post-light therapy [162]. Moreover, the antibacterial effect stops when the light is turned off. Furthermore, non-specific localization of photosensitizers could lead to light-associated toxicity [44,163,164].

## 9. Future Perspective and Directions of aPDT

The clinical need for porphyrins and aPDT already exists in hospitals worldwide and their applications have been growing rapidly in recent times. However, to improve their continual use in the clinical environment, there are many factors of aPDT that need to be considered. Some of these include more efficient light-delivery systems, the physiochemical characteristics of the porphyrins, dose to be administered, rate of drug delivery, stability and ease of application, and clearance after use. In addition, the barrier properties of the target site and patient acceptability may also have some negative impact on antibacterial PDT. Deep treatments, using porphyrins that absorb in the NIR region of the spectrum, can be achieved by two-photon PDT and/or metronomic PDT. The former is based on the development of laser technology, which allows the application of short (approx. 100 fs) laser pulses with high peak power. Instead of one, two light photons are absorbed and each photon accounts for only half of the excitation energy. Metronomic PDT is based on the application of very low doses of porphyrins combined with low rates of irradiation lasting for extended periods of time [165]. The outcome is cell death by apoptosis with minimal tissue necrosis.

The efficiency of PDT could be significantly enhanced by using nanoparticles, which can improve the photosensitizer solubility in aqueous media, its photophysical properties, and selectivity to the target tissue [166]. Targeted delivery of porphyrins can be achieved by conjugation with antibodies, engineered synthesis of molecules with specific structure, and even by attachment of porphyrins to magnetic nanoparticles. In the latter case, an externally applied magnetic field directs the PS to the area of infection [167]. Another significance of PDT that requires further investigation is its effect on the host immune system [121]. To overcome some of these challenges, PDT needs commitment and funds [168]. Information about this technique should also be disseminated by arranging aPDT-oriented workshops for chemists, physicians, biologists, and clinicians. 

## 10. Conclusions

Antibacterial photodynamic therapy as a non-toxic, non-invasive, and cost-effective method is at the forefront for the treatment of infectious diseases. It offers the potential to significantly improve the current means of antibiotic treatment and reduce potential future problems associated with antibiotic-resistant bacteria.
